# Association between high-sensitivity C-reactive protein and coronary atherosclerosis in a general middle-aged population

**DOI:** 10.1038/s41598-023-39051-3

**Published:** 2023-07-27

**Authors:** Sofia Cederström, Pia Lundman, Joakim Alfredsson, Emil Hagström, Annica Ravn-Fischer, Stefan Söderberg, Troels Yndigegn, Per Tornvall, Tomas Jernberg

**Affiliations:** 1 Department of Clinical Sciences, Danderyd Hospital, Karolinska Institutet, Stockholm, Sweden; 2 Department of Health, Medicine and Caring Sciences and Department of Cardiology, Linköping University, Linköping, Sweden; 3 Department of Medical Sciences, Cardiology, Uppsala University, Uppsala, Sweden; 4 Department of Cardiology, Sahlgrenska University Hospital, Institute of Medicine, Department of Molecular and Clinical Medicine, Sahlgrenska Academy at University of Gothenburg, Gothenburg, Sweden; 5 Department of Public Health and Clinical Medicine, Heart Centre, Umeå University, Umeå, Sweden; 6 Department of Cardiology, Clinical Sciences, Skåne University Hospital, Lund University, Lund, Sweden; 7 Department of Clinical Science and Education, Södersjukhuset, Karolinska Institutet, Stockholm, Sweden

**Keywords:** Atherosclerosis, Diagnostic markers, Epidemiology

## Abstract

Despite abundant knowledge about the relationship between inflammation and coronary atherosclerosis, it is still unknown whether systemic inflammation measured as high-sensitivity C-reactive protein (hsCRP) is associated with coronary atherosclerosis in a general population. This study aimed to examine the association between hsCRP and coronary computed tomography angiography (CCTA)-detected coronary atherosclerosis in a population-based cohort. Out of 30,154 randomly invited men and women aged 50 to 64 years in the Swedish Cardiopulmonary Bioimage Study (SCAPIS), 25,408 had a technically acceptable CCTA and analysed hsCRP. Coronary atherosclerosis was defined as presence of plaque of any degree in any of 18 coronary segments. HsCRP values were categorised in four groups. Compared with hsCRP below the detection limit, elevated hsCRP (≥ 2.3 mg/L) was weakly associated with any coronary atherosclerosis (OR 1.15, 95% CI 1.07–1.24), coronary diameter stenosis ≥ 50% (OR 1.27, 95% CI 1.09–1.47), ≥ 4 segments involved (OR 1.13, 95% CI 1.01–1.26 ) and severe atherosclerosis (OR 1.33, 95% CI 1.05–1.69) after adjustment for age, sex and traditional risk factors. The associations were attenuated after further adjustment for body mass index (BMI), although elevated hsCRP still associated with noncalcified plaques (OR 1.16, 95% CI 1.02–1.32), proposed to be more vulnerable. In conclusion, the additional value of hsCRP to traditional risk factors in detection of coronary atherosclerosis is low. The association to high-risk noncalcified plaques, although unlikely through a causal pathway, could explain the relationship between hsCRP and clinical coronary events in numerous studies.

## Introduction

The importance of inflammation as a driver of atherosclerosis is well known^1^ and C-reactive protein (CRP) and several other inflammatory cytokines have shown an association with coronary heart disease^2,3^, although mendelian randomization studies point in different directions regarding their causal effect^4–6^. Previous intervention studies have found both beneficial and neutral effects of anti-inflammatory therapy on cardiovascular events in individuals with previous myocardial infarction^7–9^, highlighting the roles of different inflammatory pathways.

Coronary artery calcium (CAC) score, calculated from computed tomography, is a marker of coronary atherosclerosis and can be used in addition to traditional risk factors for reclassification of cardiovascular risk^10^. The Multi-Ethnic Study of Atherosclerosis (MESA) studied the relationship between inflammatory biomarkers and CAC score in a population of 6814 men and women^11^. A weak association was found between CAC score and both interleukin-6 (IL-6) and fibrinogen. However, no substantial relationship could be found for the highest compared to the lowest quartile of CRP after adjustment for cardiovascular risk factors.

One major limitation of CAC score is the incapability to assess plaque burden and in particular plaque composition, including markers of vulnerability, which can be detected by contrast-enhanced coronary computed tomography angiography (CCTA). So far, studies examining the association between high-sensitivity CRP (hsCRP) and coronary atherosclerosis using CCTA have been small, including selected patients, resulting in inconsistent results^12–14^. There is a knowledge gap regarding the association between systemic inflammation, coronary atherosclerosis and degree of coronary calcification in the general population. In the Swedish Cardiopulmonary Bioimage Study (SCAPIS) more than 25,000 randomly invited individuals underwent CCTA and had plasma levels of hsCRP determined. The aim of this study was to examine the association between systemic inflammation measured as hsCRP and coronary artery atherosclerosis in a general middle-aged population, both in relation to the extent of coronary atherosclerosis and degree of coronary calcification.

## Methods

### Study population and data collection

The study group was included from SCAPIS, a national cohort with 30,154 randomly invited men and women aged 50 to 64 years from six university hospitals in Sweden (Göteborg, Linköping, Malmö/Lund, Stockholm, Umeå and Uppsala) between 2013 and 2018. The aim of SCAPIS was to study cardiovascular disease (CVD) and chronic obstructive pulmonary disease and extensive information has been collected as previously described^15^. In this sub-study, medical history, cardiac imaging, biochemistry, anthropometry and accelerometer data were used. A written consent was obtained from each study participant. The study protocol conforms to the ethical guidelines of the 1975 Declaration of Helsinki and was approved by the Swedish Ethical Review Authority (Dnr 2021-00549).

Coronary atherosclerosis was assessed at each site using identical dual-source CT scanners equipped with a Stellar Detector (Somatom Definition Flash, Siemens Medical Solution, Forchheim, Germany). An intravenous betablocker (metoprolol) to reduce heart rate and sublingual nitroglycerin to obtain vasodilatation were used. All CCTA images were analysed by local well-trained readers in a standardised manner regarding presence of atherosclerosis using Syngo.via software. Regular training sessions were arranged to assure consistency in reading and reporting. Non-contrast images were analysed and a CAC score was calculated according to Agatston^16,17^. 4712 participants not investigated with CCTA, for reasons stated in a previous report^17^, or with proximal segments not technically assessable were excluded. Venous blood (100 mL) for biochemistry analysis was collected after an overnight fast and an immuneturbidimetric method was used to analyse hsCRP by either Cobas from Roche® or Architect from Abbot®^15^. 34 individuals had missing hsCRP values and were excluded. This resulted in a study population of 25,408 individuals (Supplementary Fig. S2).

### Data analysis

Coronary artery atherosclerosis was defined as presence of plaque of any degree (1–49%, ≥ 50% diameter stenosis) or segments not assessable due to calcification in any of 18 coronary segments analysed by CCTA^18^. Severe atherosclerosis was defined as ≥ 50% diameter stenosis in any of the left main coronary artery, the proximal left anterior descending artery (LAD) or three vessel disease including ≥ 50% diameter stenosis in any of the segments in each of the LAD, the right coronary artery and the circumflex artery. The degree of calcification of plaques was studied, where plaques were defined as noncalcified if there was at least one noncalcified segment or with zero CAC score, minimally calcified if CAC score was less than 11 and calcified if CAC score was over 100. CAC score was divided in five groups based on the degree of calcification; 0, 1 to 10 (ultralow), 11 to 100 (low), 101 to 400 (moderate) and more than 400 (high) as previously described^17^.

Four categories of hsCRP were chosen. Local site values below and up to 0.65 mg/L were set to a harmonised national hsCRP variable of 0.6 mg/L, which was applied as a common lowest detection limit and used as a reference (hsCRP < 0.7 mg/L) (n = 8574). Similarly, local site hsCRP between 0.66 and 0.7 mg/L were rounded up to 0.7 mg/L. National hsCRP values above the detection limit (≥ 0.7 mg/L) were categorised in tertiles (n = 16,834).

### Statistical analysis

Continuous variables are presented using means and standard deviations (SD) and categorical variables are presented using counts and percentages. Differences between categorical variables were assessed using chi-square analysis. Logistic regression was performed to analyse the association between the different tertiles of hsCRP compared to the reference (hsCRP < 0.7 mg/L) and degree of coronary atherosclerosis and calcification. Four models were used; univariable, model 1—adjusted for age and sex, model 2—adjusted for age, sex, self-reported treatment of hypertension and/or hyperlipidemia, diabetes mellitus (self-reported or newly diagnosed in SCAPIS), and smoking habits, and model 3—model 2 and body mass index (BMI). In order to examine which covariate attenuated the association between hsCRP and coronary atherosclerosis most, each variable was also separately (one-by-one) included in a model with hsCRP, age and sex. The effect of sex was studied in a subgroup analysis. A mixed model with study site as a random effect was used to analyse the possible importance of different inclusion sites in SCAPIS in relation to hsCRP. Statistical significance was set to P < 0.05. For the statistical analysis the software SPSS version 26 was used.

## Results

### Baseline characteristics

Participant characteristics by hsCRP strata are described in Table 1. Number of participants with missing data are stated in the Supplementary Table S1. The participants, of which 50% were men, were grouped into age quartiles. A higher hsCRP was more common in subjects 61 years of age or older. Classical risk factors for cardiovascular disease such as antihypertensive medication, blood pressure levels, diabetes mellitus and smoking were more prevalent in individuals in the higher hsCRP tertiles. There was no numerical difference in hsCRP in relation to cholesterol-lowering medication, low-density lipoproteins (LDL) or creatinine. BMI, waist circumference and time being sedentary were higher in subjects with higher hsCRP tertiles. In contrast, a higher level of education, present employment and absence of economic stress was associated with lower hsCRP tertiles.

**Table 1:** Basic characteristics of the study population.

Characteristics	HsCRP (mg/L)			
	< 0.7	0.7–1.1	1.2–2.2	≥ 2.3
Sample size	8574 (33.7)	5436 (21.4)	5565 (21.9)	5833 (23.0)
Male	4176 (48.7)	2858 (52.6)	2952 (53.0)	2709 (46.4)
Age, years				
< 54	2619 (30.6)	1501 (27.6)	1418 (25.5)	1406 (24.1)
≥ 54–56	1715 (20.0)	1126 (20.7)	1118 (20.1)	1133 (19.4)
≥ 57–60	2239 (26.1)	1441 (26.5)	1443 (25.9)	1575 (27.0)
≥ 61	2001 (23.3)	1368 (25.2)	1586 (28.5)	1719 (29.5)
Cardiovascular risk factors				
Known diabetes mellitus	262 (3.0)	182 (3.3)	224 (4.0)	357 (6.1)
Newly diagnosed diabetes mellitus^a^	104 (1.2)	112 (2.1)	155 (2.8)	273 (4.7)
Elevated HbA1c	121 (1.4)	101 (1.9)	152 (2.7)	243 (4.2)
Impaired fasting glucose	919 (10.7)	668 (12.3)	782 (14.1)	976 (16.7)
Normoglycemia	7159 (83.5)	4368 (80.4)	4245 (76.3)	3979 (68.2)
Rheumatic disease	193 (2.3)	149 (2.7)	191 (3.4)	332 (5.7)
Previous cardiovascular disease^b^	137 (1.6)	74 (1.4)	64 (1.2)	67 (1.1)
Treatment^c^				
Antihypertensive medication	1187 (13.8)	875 (16.1)	1095 (19.7)	1428 (24.5)
Cholesterol-lowering medication	594 (6.9)	402 (7.4)	417 (7.5)	370 (6.3)
Diabetes medication	197 (2.3)	145 (2.7)	172 (3.1)	249 (4.3)
Clinical chemistry				
LDL, mmol/L	3.3 ± 0.9	3.5 ± 1.0	3.5 ± 1.0	3.5 ± 1.0
Non-HDL, mmol/L	3.7 ± 1.0	3.9 ± 1.1	4.0 ± 1.1	4.0 ± 1.1
Fasting glucose, mmol/L	5.5 ± 0.8	5.7 ± 0.9	5.8 ± 1.0	6.0 ± 1.3
HbA1c, mmol/mol	35.4 ± 4.7	35.8 ± 5.3	36.5 ± 5.9	38.0 ± 7.5
Creatinine, μmol/L	77.9 ± 13.5	78.2 ± 13.6	77.7 ± 13.8	76.0 ± 14.2
eGFR, ml/min/1.73 m^2^	84.7 ± 11.2	84.9 ± 11.3	85.4 ± 11.5	85.8 ± 12.0
Blood pressure, mmHg				
Systolic	123 ± 16	126 ± 17	127 ± 17	129 ± 17
Diastolic	76 ± 10	77 ± 10	78 ± 10	80 ± 11
Smoking				
Current smoker	788 (9.2)	627 (11.5)	752 (13.5)	945 (16.2)
Previous smoker	2871 (33.5)	1851 (34.1)	1985 (35.7)	2186 (37.5)
Never smoker	4658 (54.3)	2792 (51.4)	2644 (47.5)	2491 (42.7)
Pack years of cigarettes for smokers	20.0 ± 13.6	21.3 ± 14.0	23.2 ± 14.2	25.5 ± 15.6
Anthropometry				
Body mass index, kg/m^2^	24.8 ± 3.2	26.4 ± 3.5	27.7 ± 3.9	29.4 ± 4.9
Weight at 20 years of age, kg	65.2 ± 11.3	65.9 ± 11.6	66.2 ± 11.8	65.5 ± 12.0
Waist circumference, cm	88.3 ± 10.9	93.3 ± 11.2	96.8 ± 11.7	100.8 ± 13.0
Waist-hip-ratio, cm/cm	0.89 ± 0.08	0.92 ± 0.09	0.93 ± 0.09	0.94 ± 0.09
Physical activity				
Time spent sedentary, percentage of wear time	52.6 ± 10.1	53.6 ± 10.3	54.4 ± 10.2	55.6 ± 10.7
Time in moderate or vigorous activity, percentage of wear time	7.0 ± 3.5	6.6 ± 3.4	6.2 ± 3.3	5.7 ± 3.3
Sociodemographics				
University degree	4339 (51.5)	2468 (46.3)	2302 (42.3)	2179 (38.4)
Employed	7420 (86.5)	4607 (84.7)	4564 (82.0)	4495 (77.1)
Economic stress^d^	528 (6.2)	457 (8.4)	566 (10.2)	763 (13.1)

Values are in n (%) for categorical variables and mean (standard deviation) for continuous variables.

*eGFR* estimated glomerular filtration rate, *HbA1c* hemoglobin A1C, *HDL* high-density lipoprotein, *HsCRP* high-sensitivity C-reactive protein, *LDL* low-density lipoprotein.

^a^Newly diagnosed diabetes mellitus in SCAPIS is defined as fasting p-glucose ≥ 7 mmol/L or HbA1c ≥ 48 mmol/mol.

^b^Previous cardiovascular disease is defined as self-reported previous coronary artery by-pass grafting, percutaneous coronary artery intervention or myocardial infarction.

^c^Self-reported medication for hypertension, hyperlipidemia or diabetes mellitus the last two weeks.

^d^Economic stress is defined as a combination of a negative answer to the question “If you should suddenly find yourself in a situation where you had to find 2000 Euro in one week, would you manage that?” and a positive answer to the question “During the last 12 months, have you ever had difficulties in managing the regular expenses for food, rent, bills etc.?”.

### Atherosclerotic characteristics

Atherosclerotic characteristics at CCTA in relation to hsCRP tertiles and corresponding odds ratios (OR) are described in Tables 2 and 3 (univariable model). Higher hsCRP levels were associated with a larger proportion of participants with any coronary atherosclerosis, 46.5% of subjects with hsCRP ≥ 2.3 mg/L compared to 39.5% with hsCRP < 0.7 mg/L. Higher tertiles of hsCRP were also associated with a higher likelihood of having a significant coronary artery stenosis (≥ 50%), larger number of segments involved (segment involvement score) (Supplementary Fig. S1) and severe coronary atherosclerosis. Also, there was an association between hsCRP tertiles and both noncalcified, minimally calcified and calcified plaques as well as CAC score (Table 2).

**Table 2:** Atherosclerotic characteristics at CCTA in relation to hsCRP.

Characteristics	HsCRP (mg/L)				*p*-value
	< 0.7	0.7–1.1	1.2–2.2	≥ 2.3	
Coronary atherosclerosis	3385 (39.5)	2277 (41.9)	2499 (44.9)	2713 (46.5)	< 0.001
Coronary stenosis ≥ 50%	413 (4.8)	276 (5.1)	340 (6.1)	411 (7.0)	< 0.001
≥ 4 segments with atherosclerosis	954 (11.1)	657 (12.1)	740 (13.3)	872 (14.9)	< 0.001
≥ 50% stenosis in the LMCA, proximal LAD or 3VD	155 (1.8)	109 (2.0)	123 (2.2)	159 (2.7)	< 0.001
Calcified plaques	867 (10.1)	617 (11.4)	682 (12.3)	792 (13.6)	< 0.001
Minimally calcified plaques	1065 (12.4)	690 (12.7)	778 (14.0)	837 (14.3)	< 0.001
Noncalcified plaques	676 (7.9)	488 (9.0)	598 (10.7)	646 (11.1)	< 0.001
CAC score—degree of calcification					
0—none	5279 (62.6)	3235 (60.6)	3151 (57.5)	3239 (56.2)	< 0.001
1–10—ultralow	932 (11.1)	583 (10.9)	653 (11.9)	693 (12.0)	< 0.001
11–100—low	1348 (16.0)	904 (16.9)	995 (18.2)	1040 (18.0)	< 0.001
101–400—moderate	600 (7.1)	419 (7.8)	495 (9.0)	515 (8.9)	< 0.001
> 400—high	268 (3.2)	198 (3.7)	188 (3.4)	277 (4.8)	< 0.001

Values are in n (%). A chi-square test was used to generate the indicated p-values.

*CAC* coronary artery calcium, *HsCRP* high-sensitivity C-reactive protein, *LAD* left anterior descending coronary artery, *LMCA* left main coronary artery, *3VD* three vessel disease.

**Table 3:** Logistic regression models.

Univariable	Coronary atherosclerosis			Coronary stenosis ≥ 50%			Calcified plaques			Minimally calcified plaques			Any noncalcified plaque		
	OR	95% CI		OR	95% CI		OR	95% CI		OR	95% CI		OR	95% CI	
HsCRP < 0.7 (ref)	1.00			1.00			1.00			1.00			1.00		
HsCRP 0.7–1.1	1.11	1.03	1.18	1.06	0.90	1.24	1.14	1.02	1.27	1.03	0.93	1.14	1.15	1.02	1.30
HsCRP 1.2–2.2	1.25	1.17	1.34	1.29	1.11	1.49	1.24	1.12	1.38	1.15	1.04	1.27	1.41	1.25	1.58
HsCRP ≥ 2.3	1.33	1.25	1.43	1.50	1.30	1.72	1.40	1.26	1.55	1.18	1.07	1.30	1.46	1.30	1.63
Model 1															
HsCRP 0.7–1.1	1.04	0.97	1.12	0.98	0.84	1.15	1.06	0.95	1.19	1.01	0.91	1.12	1.11	0.98	1.25
HsCRP 1.2–2.2	1.14	1.06	1.23	1.15	0.99	1.34	1.11	0.99	1.24	1.12	1.02	1.24	1.33	1.18	1.49
HsCRP ≥ 2.3	1.31	1.22	1.41	1.44	1.25	1.67	1.33	1.20	1.48	1.18	1.07	1.31	1.44	1.28	1.61
Model 2															
HsCRP 0.7–1.1	1.00	0.93	1.08	0.94	0.80	1.11	1.00	0.89	1.12	1.01	0.91	1.12	1.09	0.96	1.23
HsCRP 1.2–2.2	1.07	0.99	1.15	1.11	0.95	1.29	1.03	0.92	1.15	1.11	1.01	1.23	1.28	1.14	1.45
HsCRP ≥ 2.3	1.15	1.07	1.24	1.27	1.09	1.47	1.12	1.00	1.25	1.18	1.06	1.30	1.33	1.18	1.50
Model 3															
HsCRP 0.7–1.1	0.95	0.88	1.03	0.91	0.77	1.08	0.96	0.85	1.08	0.98	0.88	1.09	1.04	0.91	1.18
HsCRP 1.2–2.2	0.98	0.90	1.06	1.05	0.90	1.23	0.96	0.85	1.08	1.05	0.95	1.17	1.18	1.04	1.33
HsCRP ≥ 2.3	0.99	0.92	1.08	1.17	0.99	1.37	1.00	0.89	1.13	1.08	0.96	1.20	1.16	1.02	1.32

Univariable	≥ 4 segments with atherosclerosis			≥ 50% stenosis in the LMCA, proximal LAD or 3VD			CAC score > 0			CAC score > 100					
	OR	95% CI		OR	95% CI		OR	95% CI		OR	95% CI				
HsCRP < 0.7 (ref)	1.00		1.00		1.00		1.00								
HsCRP 0.7–1.1	1.10	0.99	1.22	1.11	0.87	1.42	1.09	1.02	1.17	1.14	1.02	1.27			
HsCRP 1.2–2.2	1.23	1.11	1.36	1.23	0.97	1.56	1.24	1.16	1.33	1.24	1.11	1.38			
HsCRP ≥ 2.3	1.40	1.27	1.55	1.52	1.22	1.90	1.31	1.22	1.40	1.39	1.25	1.54			
Model 1															
HsCRP 0.7–1.1	1.02	0.91	1.14	1.04	0.81	1.33	1.02	0.95	1.10	1.06	0.94	1.18			
HsCRP 1.2–2.2	1.09	0.98	1.21	1.10	0.87	1.40	1.13	1.04	1.21	1.09	0.98	1.22			
HsCRP ≥ 2.3	1.36	1.23	1.51	1.45	1.16	1.82	1.28	1.19	1.37	1.32	1.18	1.47			
Model 2															
HsCRP 0.7–1.1	0.95	0.85	1.07	1.01	0.78	1.31	0.98	0.91	1.06	0.99	0.88	1.11			
HsCRP 1.2–2.2	1.01	0.90	1.13	1.10	0.86	1.41	1.04	0.97	1.13	1.01	0.90	1.13			
HsCRP ≥ 2.3	1.13	1.01	1.26	1.33	1.05	1.69	1.11	1.03	1.20	1.09	0.97	1.22			
Model 3															
HsCRP 0.7–1.1	0.90	0.80	1.01	1.00	0.77	1.30	0.93	0.86	1.01	0.95	0.84	1.07			
HsCRP 1.2–2.2	0.91	0.81	1.03	1.08	0.84	1.39	0.96	0.88	1.03	0.93	0.83	1.05			
HsCRP ≥ 2.3	0.97	0.86	1.09	1.29	0.99	1.66	0.96	0.88	1.04	0.97	0.85	1.09			

HsCRP values are in mg/L. Model 1: adjustments for age and sex; model 2: adjustments for age, sex, cardiovascular risk factors; model 3: adjustments in model 2 and body mass index.

*CAC* coronary artery calcium, *CI* confidence interval, *HsCRP* high-sensitivity C-reactive protein, *LAD* left anterior descending coronary artery, LMCA = left main coronary artery, *OR* odds ratio, *3VD* three vessel disease.

### Adjusted models

**Figure 1:**
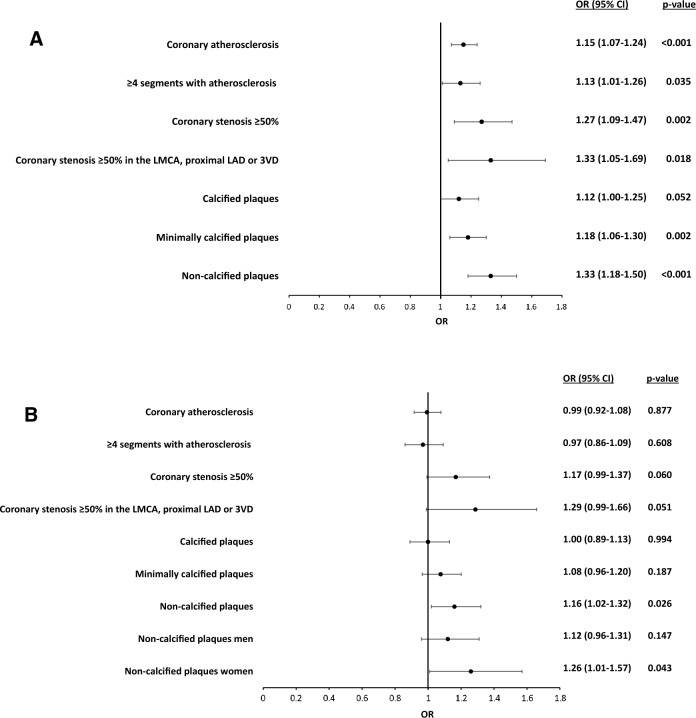
**(a)** Odds ratios (95% confidence intervals) for hsCRP ≥ 2.3 mg/L compared to hsCRP < 0.7 mg/L in model 2, including adjustments for age, sex and established cardiovascular risk factors. (**b**) Odds ratios (95% confidence intervals) for hsCRP ≥ 2.3 mg/L compared to hsCRP < 0.7 mg/L in model 3, including adjustments in model 2 and body mass index. *CI* confidence interval, *LAD* left anterior descending coronary artery, *LMCA* left main coronary artery, *OR* odds ratio, *3VD* three-vessel disease

After adjusting for age and sex (model 1), the associations between hsCRP tertiles and presence, degree and extent of coronary atherosclerosis as well as coronary calcification and noncalcified plaques remained (Table 3). After additional adjustments for traditional cardiovascular risk factors (model 2), the associations were clearly attenuated, but remained significant with a dose–response relationship between hsCRP tertiles and the degree of calcification (Table 3, Fig. 1a). In model 3 including BMI, the two highest tertiles of hsCRP remained associated with noncalcified plaques (OR 1.16, 95% CI 1.02–1.32, P = 0.026 for hsCRP ≥ 2.3 mg/L compared to the reference). For several of the other atherosclerotic characteristics including CAC score, the associations did not remain significant, although the associations for coronary diameter stenosis ≥ 50% (OR 1.17, 95% CI 0.99–1.37, P = 0.060) and severe coronary atherosclerosis (OR 1.29, 95% CI 0.99–1.66, P = 0.051) showed borderline significance for the highest tertile of hsCRP compared to the reference (Table 3, Fig. 1b). When testing each variable separately, BMI had the strongest attenuating effect on the association between hsCRP tertiles and coronary atherosclerosis (Supplementary Table S2). To omit influence of individuals with healthy coronary vessels, the relationship between hsCRP tertiles and noncalcified plaques was also analysed in the subset of individuals with detected coronary atherosclerosis, which resulted in similar estimates (OR 1.18, 95% CI 1.03–1.36, P = 0.015). In a stratified analysis, the association between hsCRP and noncalcified plaques remained in women (OR 1.26, 95% CI 1.01–1.57, P = 0.043), but not in men (OR 1.12, 95% CI 0.96–1.31, P = 0.147) (Fig. 1b). Adjustment with study site as a random effect did not change the result.

## Discussion

This is so far the largest cross-sectional study of randomly invited individuals in which the associations between hsCRP and coronary atherosclerosis detected by CCTA have been examined. We found weak associations between hsCRP and coronary atherosclerosis, calcifications and noncalcified atherosclerosis, after adjustment for age, sex and traditional risk factors. When adjusting also for BMI, the associations were further attenuated, but still significant for noncalcified plaques.

The association between CRP and cardiovascular events is well established^3,19^, but the possible relationship between CRP and presence of coronary atherosclerosis is less studied and has mainly focused on the degree of coronary calcification as measured by CAC score. An analysis of 321 individuals with no prior CVD from the Framingham Heart Study (FHS) found associations between high CRP levels and Agatston score, but was limited to CRP in samples collected 4–8 years before CCTA^12^. In contrast, several small studies^14,20,21^ and the large MESA study^11^, did not show any association between CRP and CAC score after adjustment for traditional risk factors including obesity, which was confirmed in the present study. Although known to be associated with coronary atherosclerosis and implicate a low risk of CVD events in individuals with a low score^22^, the CAC score has limitations. A recent analysis from SCAPIS found that zero CAC score did not exclude atherosclerosis and could even include individuals with significant coronary artery stenosis, left main coronary artery stenosis, proximal LAD stenosis and three-vessel disease^17^, why the search for markers of coronary atherosclerosis should not be restricted to investigating CAC score. Research regarding CRP and coronary atherosclerosis in samples from the general population is sparse. One study of 2554 patients with angina found a positive association between CRP and both the number and severity of atherosclerotic lesions at coronary angiography in a unadjusted analysis, although correlation coefficients were low^13^. This is in line with our results where hsCRP was associated with any coronary atherosclerosis in the univariable model.

In our study, the association between hsCRP and coronary atherosclerosis was weak, but still evident after adjusting for the traditional risk factors included in the Systemic Coronary Risk Estimation 2 (SCORE2)^23^. However, after addition of BMI to the model, the associations between hsCRP and presence, degree and extent of coronary atherosclerosis did not remain (Table 3, Fig. 1b). When each possible confounder was added separately to model 1, the OR was most influenced by BMI, which suggests that the relationship between hsCRP and coronary atherosclerosis is driven by systemic inflammation in obese study subjects. This is supported by previous knowledge where elevated CRP has been associated with an increased BMI^24^.

Another finding from the present study is the association between elevated CRP and noncalcified plaques detected by CCTA. The importance of plaque morphology for the risk of rupture has previously been highlighted. Noncalcified plaques with a larger necrotic core and a thin fibrous cap result in a coronary plaque more prone to rupture^25,26^. Presence of noncalcified non-obstructive plaques on CCTA have been proposed to better predict all-cause mortality compared with calcified plaques^27^ and was recently found to be a stronger predictor of cardiovascular events than both CAC score, degree of stenosis and traditional risk factors^26,28^. Also, statin treatment seems to slow progression of noncalcified plaques and reduce noncalcified plaque volume^29,30^. Recently, elevated CRP was associated with high-risk plaque features as presence of low attenuation plaques (LAP), defined as < 30 Hounsfield units (HU) in 524 middle-aged patients without known CVD and with clinical indication for CCTA, after adjustment for cardiovascular risk factors. However, no relationship was found between CRP and quantitative measures as LAP volume, noncalcified plaque volume (< 150 HU) or total plaque volume^31^. Although diverging results, the authors suggested that systemic inflammation is related to presence of high-risk atherosclerosis, which is in accordance with our study. This is also supported by the reduction of both recurrent cardiovascular events and hsCRP after treatment with canakinumab^7^. Somewhat in conflict with our finding, Rubin et al. found that CRP ≥ 2 mg/L was associated with presence of both atherosclerotic and mixed plaques, in contrast to noncalcified or calcified plaques in asymptomatic individuals in a model adjusted for cardiovascular risk factors^32^.

Although our observational study design does not allow for any conclusions regarding mechanisms for the association between hsCRP and plaque morphology characteristics, one could speculate that high hsCRP reflects an increased risk of coronary atherosclerosis through systemic inflammation from adipose tissue, where CRP is a downstream biomarker of IL-6 and other proinflammatory cytokines^33^, as well as acts as an independent marker for a more vulnerable plaque. A meta-analysis including 47 mendelian randomization studies with 194,418 participants previously studied single nucleotide polymorphisms of the CRP gene and found no association between genetically elevated CRP protein levels and coronary heart disease^4^. Therefore, elevated hsCRP is likely a proxy for the systemic inflammation in participants with noncalcified plaques and not involved in a causal pathway. In contrast, similar mendelian randomization studies have suggested that the IL-6 receptor might have a causal role in coronary artery disease and could be a possible drug target^5,6^.

In a subgroup analysis, hsCRP showed a small, but significant association with noncalcified plaques in women, but not in men. This result should be interpreted with caution. However, sex differences with higher CRP levels in women compared to men have previously been found in both the MESA cohort^34^ and a group with carotid stenosis from the FHS^35^. Also, hormonal effects with increased CRP in men on estrogen treatment have been shown^36^, which could be a possible explanation for the above findings. Yet, the corresponding impact on risk of CVD is not necessarily the same for men and women^37^.

This study has several strengths; (1) the results are from a very large population-based cohort of middle-aged men and women with (2) standardised collected blood sampling and (3) quality-checked CCTA. The study also has several limitations. First, hsCRP was just measured once, though previously shown to be a stable biomarker with no obvious circadian variation^38^. Second, although randomly invited, the study group may suffer from selection bias. Third, we lacked detailed information about pharmacological treatment. We only had self-reported information regarding treatment of hypertension and hyperlipidemia, which may cause information bias. Fourth, many statistical tests were performed to examine the association between hsCRP and different outcomes in different models. However, the main aim was to analyse the relationship between hsCRP and presence of coronary atherosclerosis and degree of calcification after adjustment of all relevant risk factors, why no correction for multiple testing was made. Fifth, although SCAPIS is a very large randomly invited population-based cohort and the results are likely applicable in other populations, the use of an external validation cohort would have strengthened the result. Sixth, the study design does not allow for any conclusion regarding mechanisms for the associations between hsCRP and coronary atherosclerosis. Nevertheless, data of other inflammatory biomarkers, for example IL-6, would have added information about the role of systemic inflammation in coronary atherosclerosis.

In conclusion, in this large population-based cohort we found elevated hsCRP to be only weakly associated with presence of coronary atherosclerosis after adjustment for traditional cardiovascular risk factors and thereby not useful to identify individuals who may benefit from CCTA. This association was largely explained by BMI, indicating an important interplay between adipose tissue, systemic inflammation and coronary atherosclerosis. There was also an association between hsCRP and presence of coronary atherosclerosis with noncalcified plaques, which may explain the known associations between hsCRP and clinical events.

## Supplementary Information

Supplementary Information.

## Data Availability

The datasets generated during and analysed during the current study are not publicly available due to legal reasons, but are available from the corresponding author on reasonable request.
