# Supplementary material for: Association between high-sensitivity C-reactive protein and coronary atherosclerosis in a general middle-aged population
Source: Sci Rep. 2023 Jul 27;13:12171. doi: 10.1038/s41598-023-39051-3 (PMC10374905; doi:10.1038/s41598-023-39051-3)
Supplement: Supplementary file 1 — Supplementary Information. [file 41598_2023_39051_MOESM1_ESM.pdf]

Supplementary information for the manuscript: Association between high-sensitivity C-reactive protein and coronary atherosclerosis in a general middle-aged population

Sofia Cederström*, Pia Lundman, Joakim Alfredsson, Emil Hagström, Annica Ravn-Fischer, Stefan Söderberg, Troels Yndigegn, Per Tornvall, Tomas Jernberg

Figure S1. Proportion of participants by segment involvement score per hsCRP strata

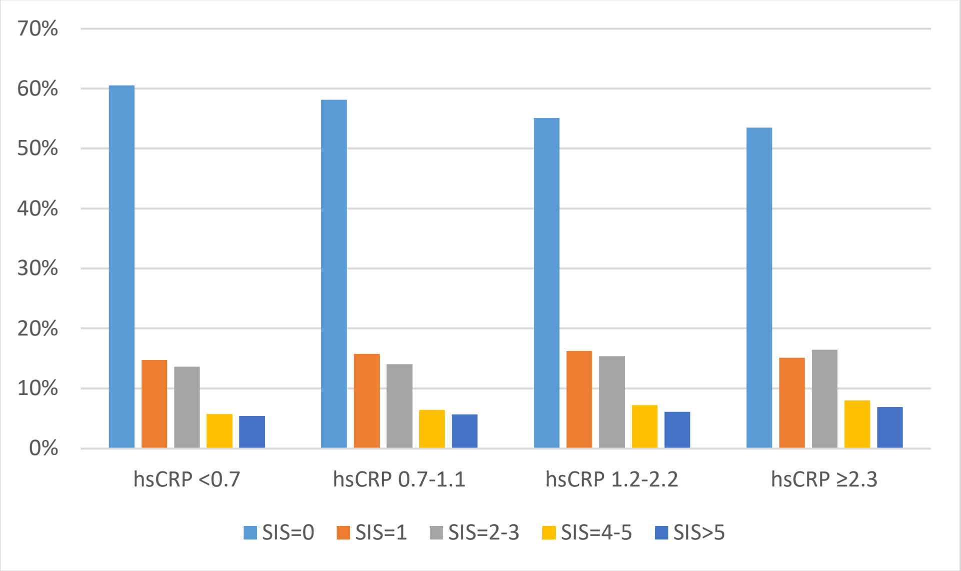

Proportion of participants (y-axis) by number of segments involved per hsCRP strata in mg/L (x-axis). hsCRP=high-sensitivity C-reactive protein, SIS=segment involvement score

Figure S2. Flow chart of the study population

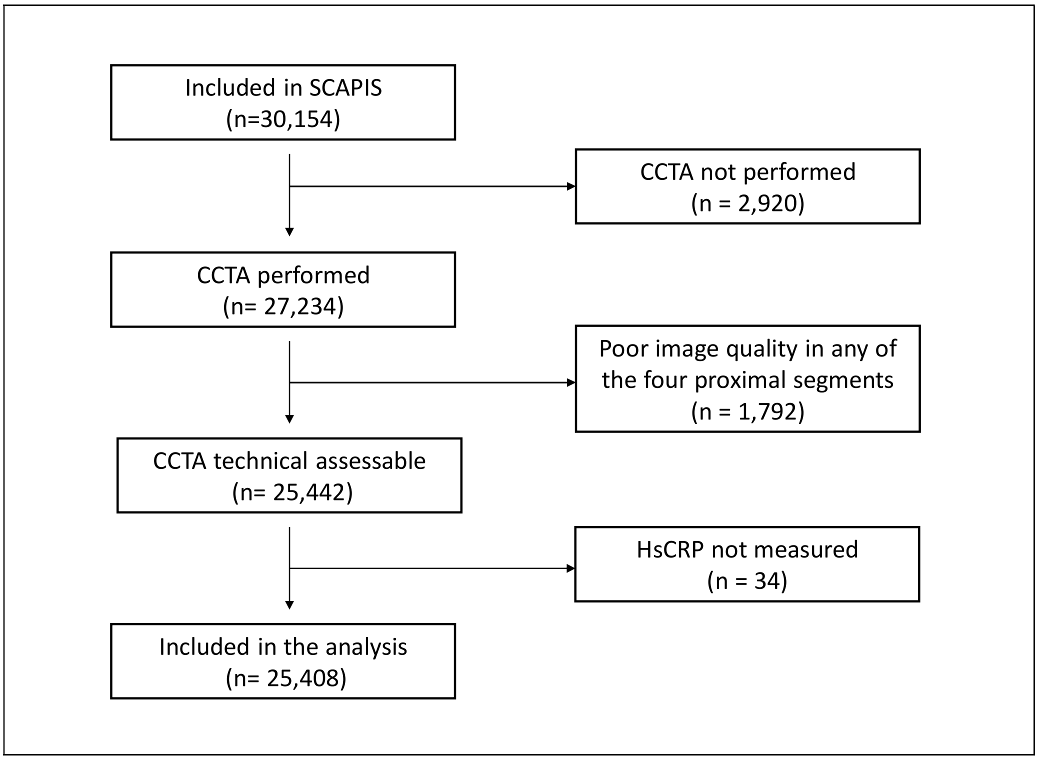

Number of study participants included and reasons for exclusion. CCTA=coronary computed tomography angiography, HsCRP=high-sensitivity C-reactive protein, SCAPIS=Swedish Cardiopulmonary Bioimage Study.

Table S1. Number of participants with missing data

**Cardiovascular risk factors**	**Number of participants with missing data (%)**
Gender	0 (0%)
Age	0 (0%)
Systolic blood pressure	4 (0.0%)
Diastolic blood pressure	6 (0.0%)
Smoking status	818 (3.2%)
Glycaemic status	26 (0.1%)
Previous CABG or PCI	820 (3.2%)
Previous myocardial infarction	820 (3.2%)
Rheumatic disease	820 (3.2%)
**Treatment**	
Antihypertensive medication	830 (3.3%)
Cholesterol-lowering medication	835 (3.3%)
Diabetes medication	831 (3.3%)
**Clinical chemistry**	
LDL	127 (0.5%)
Non-HDL	19 (0.1%)
Fasting glucose	2,160 (8.5%)
HbA1c	75 (0.3%)
Creatinine	10 (0.0%)
eGFR	10 (0.0%)
**Anthropometry**	
Body mass index	0 (0%)
Weight at 20 years of age	1,947 (7.7%)
Waist circumference	8 (0.0%)
Waist-hip-ratio	15 (0.1%)
Accelerometer data	867 (3.4%)
**Sociodemographics**	
Highest completed level of education	596 (2.3%)
Present occupation	658 (2.6%)
Ability to find 2,000 Euro in one week for unforseen events	1,070 (4.4%)
Difficulties managing regular expenses during the last 12 months	820 (3.2%)

Values are in n (%). CABG= coronary artery by-pass grafting, eGFR=estimated glomerular filtration rate, HbA1c=hemoglobin A1C, HDL=high-density lipoprotein, LDL=low-density lipoprotein PCI=percutaneous coronary artery intervention

Table S2. Odds ratios for model 1 including each covariate one-by-one.

	Coronary atherosclerosis		
**Model 1**	**OR**	**95% CI**	
HsCRP <0.7 (ref)	1.00		
HsCRP 0.7-1.1	1.04	0.97	1.12
HsCRP 1.2-2.2	1.14	1.06	1.23
HsCRP ≥2.3	1.31	1.22	1.41
**Model 1 + treatment of hypertension**			
HsCRP 0.7-1.1	1.02	0.95	1.10
HsCRP 1.2-2.2	1.11	1.03	1.19
HsCRP ≥2.3	1.22	1.13	1.31
**Model 1 + treatment of hyperlipidemia**			
HsCRP 0.7-1.1	1.03	0.96	1.11
HsCRP 1.2-2.2	1.15	1.07	1.24
HsCRP ≥2.3	1.32	1.23	1.42
**Model 1 + diabetes mellitus**			
HsCRP 0.7-1.1	1.03	0.96	1.11
HsCRP 1.2-2.2	1.13	1.05	1.21
HsCRP ≥2.3	1.26	1.17	1.36
**Model 1 + smoking**			
HsCRP 0.7-1.1	1.02	0.94	1.09
HsCRP 1.2-2.2	1.11	1.03	1.19
HsCRP ≥2.3	1.23	1.15	1.33
**Model 1 + systolic blood pressure**			
HsCRP 0.7-1.1	1.01	0.94	1.09
HsCRP 1.2-2.2	1.10	1.02	1.18
HsCRP ≥2.3	1.22	1.14	1.32
**Model 1 + non-HDL**			
HsCRP 0.7-1.1	1.00	0.93	1.08
HsCRP 1.2-2.2	1.08	1.01	1.16
HsCRP ≥2.3	1.25	1.16	1.34
**Model 1 + body mass index**			
HsCRP 0.7-1.1	0.96	0.89	1.03
HsCRP 1.2-2.2	0.99	0.92	1.07
HsCRP ≥2.3	1.04	0.96	1.13

CI=confidence interval, HDL=high-density lipoprotein, HsCRP=high-sensitivity C-reactive protein, OR=odds ratio
